# The state of the art in assessing mental fatigue in the cockpit using head-worn sensing technology

**DOI:** 10.3389/fnrgo.2025.1673268

**Published:** 2026-01-12

**Authors:** Anneke Hamann, Carmen van Klaren, Rolf Zon, Frédéric Dehais, Nils Carstengerdes, Maykel van Miltenburg, Kalou Cabrera Castillos

**Affiliations:** 1 Institut für Flugführung, Deutsches Zentrum für Luft- und Raumfahrt e.V. (DLR), Braunschweig, Germany; 2 Royal Netherlands Aerospace Centre (NLR), Department of Safety and Human Performance, Amsterdam, Netherlands; 3 ISAE-SUPAERO, Fédération ENAC-ISAE SUPAERO-ONERA, Center of Neuroergonomics, Toulouse, France

**Keywords:** aviation, EEG, eye-tracking, fNIRS, incapacitation, mental fatigue, physiology, pilots

## Abstract

Mental fatigue is an important construct for aviation as it can impact pilots' performance. However, its assessment has been and still is challenging. Most research done in this field is based on basic laboratory experiments, and the measurement methods in use have certain limits one needs to overcome in order to apply them in a cockpit. In this review, we present an overview of research on mental fatigue, its assessment and the gap between fundamental research and its application in aviation. We provide an overview over classical experimental paradigms for mental fatigue induction and subjective measures, as well as advanced head-worn sensing technologies (or such that target head and face), namely electroencephalography (EEG), functional near-infrared spectroscopy (fNIRS) and eye-tracking. For each measure, we discuss limitations and open challenges. Finally, we draw conclusions on the feasibility of integrating the measurements into the cockpit. We also highlight gaps that future research needs to bridge.

## 1 Introduction: the future of aviation and detecting pilot incapacitation

Commercial aviation is steadily moving toward deeper automation to support the flight crew. Two emerging concepts of operation formalize a reduced on-board crew: Reduced-Crew Operations (RCO)—with one pilot actively flying during low-demand segments such as cruise while the other rests for longer than traditional controlled rest—and Single-Pilot Operations (SiPO)—with a single pilot from take-off to landing, continuously supervising advanced assistance systems. In both cases, automation shifts the pilot's role from active manipulation to continuous supervision and decision authority. The human remains ultimately responsible for detecting anomalies and managing deviations from the plan. Accordingly, safety hinges on the pilot's ability to sustain alertness and intervene effectively across the entire flight (Fatigue Countermeasures Working Group, 2018).

Operating alone elevates the importance of detecting drifts from an optimal state—from subtle degradations to full incapacitation. Incapacitation spans partial forms (e.g., injuries limiting control inputs) to total loss of function (e.g., unconsciousness) and includes subtle states not immediately observable to others, such as headache, blurred vision, and degraded physiological or cognitive states (stress, overload, mental fatigue). For a comprehensive overview of the causes of different types of cognitive incapacitation and its effects on pilots, see Deniel et al. (2025) and Causse et al. (2025). Among potential risks of cognitive incapacitation, one is particularly salient in automation-centric cockpits: the out-of-the-loop (OOTL) phenomenon. Long stretches of nominal, low-demand monitoring can foster disengagement and erode situation awareness, setting the conditions for OOTL (Berberian et al., 2017; Hopstaken et al., 2015). While overload, sudden stress, and startle can precipitate OOTL, the risk also rises when a lone pilot supervises a highly automated RCO/SiPO cockpit—either through cumulative time-on-task in monotonous vigilance or through sustained demanding episodes that deplete cognitive resources and induce mental fatigue (MF) (Charbonnier et al., 2016; Grandjean, 1979; van Weelden et al., 2022). MF can be defined as an acute, non-pathological state induced by task demands. It lies between high alertness and sleepiness, marked by subjective weariness, reduced alertness, and the desire to disengage (Grandjean, 1979). Unlike sleepiness it is reversible with breaks (Charbonnier et al., 2016; Lal and Craig, 2001; Okogbaa et al., 1994; Shen et al., 2006; van Weelden et al., 2022). More information on the biological origins of mental fatigue can be found in Pessiglione et al. (2025).

As mental fatigue (MF) increases, core executive functions degrade in well-documented ways. MF impairs task control, planning, and preparation, yielding slower and less effective action selection (Lorist et al., 2000). It reduces attentional resources needed to detect and process unexpected events such as auditory alarms (Dehais et al., 2018) and degrades inhibitory control and error/action monitoring (Boksem et al., 2005, 2006). MF is also associated with working-memory decrements that constrain guidance of ongoing behaviour (Borragán et al., 2017; Karthikeyan et al., 2022). Decision processes change under MF, with measurable shifts in risk preference and feedback processing (Jia et al., 2022). In aviation-like, prolonged operations, MF and cognitive performance co-vary over mission time, producing less flexible responsiveness to dynamic demands (Rosa et al., 2022). In sum, evidence shows that rising MF degrades attention control, inhibition, error monitoring, planning/action execution, working memory, and decision-making. These impairments undermine system monitoring and adaptive responding, leading to poorer situation awareness (Zhou et al., 2023) and a reduced ability to adapt to external contingencies (e.g., adverse weather, failures) and to take appropriate decisions (e.g., go-around/abort landing) when required.

Framing cognitive incapacitation as a continuum—rather than a binary state—highlights the value of detecting and forecasting a pilot's drift toward impairment early, so mitigation can be timely and adaptive (Paz Gonçalves Martins et al., 2021; Reston et al., 2002). In RCO/SiPO, the usual cross-check from a second crewmember is missing; a lone pilot may not perceive their own decline, which strengthens the case for onboard incapacitation monitoring tailored to single-operator supervision (Deniel et al., 2025). To be effective in this context, monitoring must be non-invasive, unobtrusive, and sensitive to gradual, covert changes—especially when highly automated flight reduces the frequency of pilot inputs. Control-input monitoring can flag illogical actions, but risks missing slow, subclinical drifts when interventions are rare. By contrast, head-/face-oriented sensing offers continuous, real-time windows into cognitive state: EEG provides direct neural markers with millisecond resolution; fNIRS captures cortical haemodynamics with relative robustness to electrical noise; and eye-tracking indexes MF through blinks, saccades, dwell time, pupil dynamics. Each modality brings distinct strengths and integration challenges for the cockpit, motivating a progressive, operationally viable transition from lab validation to real-time assistance (Aricò et al., 2018; Peysakhovich et al., 2018).

There have been past attempts to review and structure the literature around MF and ways of assessing it via (neuro-) physiological and behavioural measures. For example, reviews both from fundamental (Tran et al., 2020) and driving research (Stancin et al., 2021) exist that discuss the usefulness of different EEG indices in assessing MF. In the context of driving, Lohani et al. (2019) discussed practical implications and challenges of bringing multiple neurophysiological, peripheral physiological and behavioural assessment methods to real-world applications. A recent review on MF assessment via peripheral physiology and eye-tracking with a distinct focus on pre- vs. post-fatigue assessments included only three studies focused on fixed-wing aircraft (Dickens et al., 2024). The aviation literature on continuous, cockpit-ready MF monitoring, however, remains comparatively sparse and the findings are hard to compare between different experimental paradigms and varying fidelity (van Weelden et al., 2022). We therefore begin our review with established laboratory paradigms for inducing MF and discuss how their mechanisms map to aviation-relevant demands. We then review subjective, behavioural, and physiological assessment methods of MF with emphasis on head-worn/face-targeted methods—EEG, fNIRS, and eye-tracking—, empirical findings, and limitations for real-time, in-cockpit use. We close by outlining integration challenges (comfort, certification, artefacts, online processing, individual calibration), gaps between lab and flight decks, and a staged roadmap toward robust, multimodal pilot-state monitoring that can safeguard single-operator operations against OOTL and MF hazards.

## 2 Paradigms for inducing mental fatigue

Most of what we know about MF has been established in controlled, foundational paradigms designed to isolate effects on primary executive functions. Detailing these paradigms is essential here because the neurophysiological (EEG, fNIRS) and eye-tracking measurements reviewed in the following sections have largely been developed, validated, and interpreted within these task frameworks. Although these tasks are not “aviation” *per se*, they provide the mechanistic bedrock for understanding MF and for selecting candidate markers that can transfer to cockpit contexts. The utilized tasks follow at least one of two characteristics: they are cognitively challenging and require sustained attention and mental effort, and/or they are of considerable length and drain cognitive resources through time on task. In some experiments, participants undergo a sequence of different tasks to maximise the induced MF at the end of the experiment. In others, one long, monotonous MF-inducing task is combined with a shorter second task before and after, to allow for a pre- and post-MF induction assessment. Although not aviation *per se*, these paradigms map directly to cockpit-relevant functions: ability to remain alert for rare events, alarm/callout detection, response inhibition under monotony, and keeping and updating goals under load—all critical in RCO/SiPO supervision. In this section, we provide an overview of commonly used experimental paradigms for MF induction.

In cognitive psychology, various tasks and paradigms have been designed to investigate MF, aiming to replicate real-world demands or explore the cognitive processes underlying sustained attention. Thus, tests aiming at attention and continuous monitoring of stimuli are widely used to induce MF. The Psychomotor Vigilance Task (PVT; Dinges and Powell, 1985) is a well-established paradigm, commonly used to measure reaction times in response to visual stimuli presented at random intervals (Drummond et al., 2005). Unlike tasks involving complex decision-making, the PVT isolates the effects of fatigue on simple reaction times, making it particularly valuable for sleep research and studies of MF. It is highly sensitive to lapses in attention caused by sleep deprivation, prolonged cognitive effort, or other fatigue-inducing factors. The Sustained Attention to Response Task (SART; Robertson et al., 1997) offers a different approach to studying vigilance and cognitive control (Durantin et al., 2015). In SART, participants are presented with a rapid sequence of stimuli, such as numbers, and must withhold responses to rare target stimuli while responding to frequent non-targets. This task is particularly effective in examining the relationship between sustained attention, impulsivity, and mind-wandering, offering insights into how lapses in vigilance occur during repetitive tasks. Another widely used paradigm is the Continuous Performance Test (CPT; Rosvold et al., 1956), which requires participants to respond selectively to target stimuli presented in a stream of distractors (Bearden et al., 2004). The CPT measures vigilance and response inhibition and is often used in clinical settings to assess attention deficits, such as in individuals with ADHD or neurological disorders. Variants of the CPT include the AX-CPT, which introduces contextual cues to investigate sustained attention under more complex conditions. Eventually, one of the most iconic paradigms is the Mackworth Clock Task (MCT), introduced by Mackworth (1948) as an experimental simulation of long-term monitoring by radar operators in the British Air Force during World War II. In this task, participants monitor the movement of a clock hand and must detect infrequent and unpredictable target events, such as the clock hand skipping a step (Martel et al., 2014). It simulates real-world monitoring tasks, such as radar operation, making it ideal for assessing the sustained attention required in high-stakes environments like aviation or surveillance.

A second type of tasks uses the mechanism of cognitive control to elicit demand and produce MF over time. For example, the Go/No-Go Task is used to study attention and inhibitory control, where participants respond to “Go” stimuli and withhold responses to “No-Go” stimuli (Shaw et al., 2013). This task is frequently employed to examine how MF and sustained attention demands affect response inhibition and decision-making over time. Another such task is the Stroop task (Stroop, 1935) in which the participant is shown a word for a colour (such as “red”) with either matching or mismatching font colour. The participant has to correctly name the colour the word is printed in while ignoring its meaning. Similarly, the Flanker task (Eriksen and Eriksen, 1974) requires the participant to respond to a stimulus while ignoring flanking distractors. Another cognitive control test is the Sternberg task, which requires the participant to memorize target letters presented in one colour while ignoring distractor letters presented in another colour. All these tasks require selective attention and cognitive control, and prolonged execution induce MF.

Continuous working memory load is a third way of inducing MF. One example of this is the n-back task. The n-back paradigm goes back to Kirchner (1958), but has since been used in countless variations (Owen et al., 2005). The basic principle is as follows: a participant is presented with a continuous series of stimuli, often visual or auditory like letters. For each stimulus, they have to decide if it is the same as the one presented *n* steps before (e.g., 1 before in a 1-back condition, 2 before in a 2-back condition etc.) by pressing a button. Research has shown that even prolonged 1-back task execution can lead to MF due to the monotonous, repetitive nature of the task (Grissmann et al., 2017). Another way to induce MF by increasing working memory load is the Uchida–Kraepelin test (U–K test). Here, participants need to perform serial addition tasks as fast and accurate as possible, which requires considerable mental effort and sustained attention. Additionally, these working memory paradigms can be combined with classical tasks such as an interfering secondary task (e.g., an odd/even decision task) to increase mental effort and accelerate resource depletion, thereby inducing a higher and faster onset of MF (Borragán et al., 2017). One key advantage of such paradigms is their potential use as a preparatory phase before a primary task—for example, as a warm-up before a flight simulator session—with the specific goal of pre-inducing mental fatigue in order to assess its impact on operational performance.

While this section does not provide a complete list of tasks and paradigms, it illustrates that most of the research done on MF, its development and behavioural and physiological correlates, is based on these simple laboratory tasks. Research on applied tasks often uses time on task (i.e., prolonged task execution) to induce MF, or combine task execution with prior sleep deprivation (Ahn et al., 2016; Khan et al., 2015). Moreover, in realistic tasks without the ability to control all confounding factors, cognitive states may interact (Roy et al., 2013). In sum, even though the discussed paradigms and cognitive functions are relevant for pilots and cockpit tasks and provide the foundation for research on MF, the findings based on these basic research paradigms should not be projected onto applied and real-world settings without careful consideration and empirical evaluation.

## 3 Measures of mental fatigue

### 3.1 Common subjective measures of mental fatigue

The easiest way of assessing MF is by collecting subjective ratings. These measures usually have very little technical requirements, are easy to administer and seem rather face-valid both to participants and experimenters because MF can be asked about directly. Thus, there is a broad range of scales available to capture different aspects of MF (and related concepts) across various domains.

One widely used tool is the Visual Analogue Scale (VAS), which measures self-reported levels of fatigue on a simple linear scale, offering a quick and effective way to assess subjective fatigue (Shahid et al., 2012). The Karolinska Sleepiness Scale (Akerstedt and Gillberg, 1990) is a one-item measure often used to assess MF. While originally designed for sleep research, the first half of the scale can also capture shifts in attention towards feeling mentally fatigued. The KSS thereby demonstrates that MF can be considered somewhere in the middle of the continuum between attentive and sleepy. Based on the KSS, the F-ISA (Hamann and Carstengerdes, 2020) is a short and face-valid one-item MF measurement that is designed to only capture MF without extending towards sleepiness. Another notable instrument is the Chalder Fatigue Questionnaire (CFQ), a validated tool designed to measure the severity of perceived fatigue. This scale has been extensively applied in neuroergonomics to examine the interplay between mental workload and fatigue (Jackson, 2015; Young et al., 2015). The Multidimensional Fatigue Inventory (MFI-20) is another comprehensive tool that evaluates multiple dimensions of fatigue, including general fatigue, physical fatigue, MF, reduced activity, and reduced motivation (Mehta and Parasuraman, 2013; Smets et al., 1995). Similarly, the Mental Fatigue Scale (MFS) has been shown to effectively quantify MF in specific populations. This scale assesses cognitive and physical dimensions of fatigue and has demonstrated satisfactory statistical properties (Díaz-García et al., 2021).

Additionally, the NASA Task Load Index (NASA-TLX), though originally designed to assess workload, has been adapted to measure MF in operational and experimental settings. Its ability to quantify task-related fatigue through subjective ratings of mental demand, physical demand, and temporal demand makes it a valuable tool in human factors research (Hart and Staveland, 1988). The Dundee Stress State Questionnaire (DSSQ) also contributes to the assessment of MF, as it provides insights into stress and fatigue levels by evaluating subjective task engagement and distress (Matthews et al., 1999).

The development and application of these subjective scales highlight their importance in capturing the nuanced experiences of MF. These tools play a vital role in advancing our understanding of fatigue effects on cognitive and physical functioning and contribute significantly to developing targeted strategies for mitigating its impact in operational settings. Nevertheless, subjective measures suffer from certain drawbacks that limit their application to real-world settings. Such data can only be gathered by either interrupting the task to get a real-time estimate, or by applying the scales after the task and assessing MF in retrospect. Moreover, the validity of the measures is limited by the participants' ability for introspection, their willingness to give true answers, and other sources of bias like individual response styles (Weijters et al., 2010a,b). As such, subjective measures lack the unobtrusiveness, objectivity and ability to measure continuously which one would want for a MF assessment method for cockpit applications. Thus, when trying to detect incapacitation due to MF in pilots, researchers need to look beyond subjective measures and find more suitable candidate measurements, such as the physiological and behavioural measurements described in the following sections.

### 3.2 Electroencephalography

Electroencephalography (EEG), which measures electrical activity generated by cortical pyramidal neurons, is a vital tool for monitoring brain activity in real-world environments (Gramann and Plank, 2019). Wet-electrode high-density EEG (HD-EEG) systems remain the gold standard due to their superior signal quality, noise-reduction capabilities, and source localization accuracy. However, these systems are bulky and require extensive setup times, which limits their practical use in operational settings. To address these challenges, a new generation of more portable systems with fewer electrodes (e.g., 32 or 16) or wireless semi-dry and dry-electrode setups have been developed. These systems provide greater mobility and faster setup times but are often associated with lower signal-to-noise ratios (Di Flumeri et al., 2019).

#### 3.2.1 Measuring mental fatigue using EEG

##### 3.2.1.1 Event related potentials

Time-domain analyses, such as event-related potentials (ERPs), enable the examination of stimulus-locked brain responses (e.g., neural response to the onset of auditory alarms), revealing insights into perceptual, attentional, and motor processes as well as related mental effort (Ghani et al., 2020). As an objective and discrete measure, ERPs serve as a robust indicator of MF, reflecting alterations in cognitive processing as fatigue develops. These measurements rely on precise synchronization between the presented stimuli and the EEG recordings, ensuring the accuracy of the data. Key ERP features, such as amplitude and latency, are particularly informative. A reduction in the amplitude of ERPs is often associated with decreased cognitive resource allocation, while increased latencies reflect slowed neural processing, both of which are indicative of increased MF.

##### 3.2.1.2 Spectral analyses

In contrast to time-domain analyses, frequency-domain analyses decompose EEG signals into distinct frequency bands—delta (1–4 Hz), theta (4–8 Hz), alpha (8–12 Hz), beta (13–30 Hz), and gamma (30–150 Hz)—providing valuable insights into underlying neural and cognitive states, such as MF (Borghini et al., 2014). Time-frequency analyses enable continuous tracking of brain dynamics, while advancements in source localization techniques (Mullen et al., 2013) enhance the spatial resolution of EEG, allowing researchers to study neural network dynamics in real-world contexts, offering promising opportunities for neuroergonomics research.

##### 3.2.1.3 Steady State Visual Evoked Potentials (SSVEPs)

SSVEPs are brain responses elicited by periodic visual stimulation, typically in the form of flickering lights or patterned stimuli presented at a constant frequency (Norcia et al., 2015). These evoked responses appear as frequency-locked oscillatory activity in the EEG, matching the stimulation frequency and its harmonics. SSVEPs are particularly advantageous because they are robust, require minimal cognitive effort from the participant, and can be detected with relatively short calibration times. Crucially, SSVEPs are highly modulated by attention. When multiple stimuli flickering at distinct frequencies are presented (e.g., one at 11 Hz and another at 12 Hz), the brain response is selectively enhanced for the attended frequency while being suppressed for the ignored one. This feature allows SSVEPs to serve as an implicit and continuous measure of attentional focus and MF. Previous studies by Silberstein et al. (1990) and O'Connell et al. (2009b) have demonstrated that SSVEP responses are significantly affected by vigilance declines, with reductions in SSVEP amplitude corresponding to attentional lapses. However, SSVEPs also present certain drawbacks due to their intrusiveness: the repetitive visual flashes can induce visual fatigue and distract participants from the primary task. To mitigate these effects, recent studies have proposed solutions such as reducing flash brightness (Ladouce and Dehais, 2024) or using contrast-based textures (Dehais et al., 2024), both set to near-threshold (periliminal) levels, making them barely perceptible to participants—while still preserving sufficient intensity to elicit robust brain responses.

#### 3.2.2 Empirical findings

EEG has been extensively applied to investigate MF and attentional fluctuations, providing valuable insights into the neural mechanisms underlying cognitive performance. Tasks such as the SART and the PVT have revealed associations between task-unrelated thoughts, EEG frequency band changes, and performance lapses (Groot et al., 2021; Molina et al., 2019; Torkamani-Azar et al., 2020). Similarly, the MCT has been used to study vigilance decrements, demonstrating relationships between changes in frontal theta and parietal theta power and performance declines (Boksem et al., 2005; Esposito et al., 2022; Wascher et al., 2014). Several studies have identified specific neurophysiological markers that signal impending lapses in vigilance. For example, O'Connell et al. (2009a) reported increased alpha power in the right inferior parietal cortex up to 20 s before errors occurred during a continuous temporal expectancy task, suggesting its potential as a neural indicator of approaching lapses. In a prolonged Flanker task, Eichele et al. (2010) observed a gradual decrease in N2 amplitude several trials before errors. Shou et al. (2015) found that pre-stimulus alpha activity predicted errors in a prolonged colour-word matching Stroop task. Martel et al. (2014), using the MCT, observed distinct neural patterns preceding lapses. These included increased alpha power about 10 s before a missed target, likely indicating a shift toward internally focused attention. They also reported a reduction in the P3 component, which reflects diminished attentional resource allocation. Importantly, this reduction occurred in response to events up to 5 s before lapses, suggesting progressive task disengagement. More recently, Ladouce et al. (2025) demonstrated the potential of using SSVEP to tag MF and predict attentional errors. They applied low-luminance, minimally intrusive 14 Hz flickers during a 45-min MCT. SSVEP amplitude decreased prior to lapses of attention, providing a predictive neural marker for attentional disengagement. Unlike traditional alpha and theta markers, SSVEPs offered a temporally stable and specific measure of vigilance that was unaffected by prolonged task engagement. These results highlight the suitability of SSVEPs for real-time tagging of MF in sustained attention tasks, which is particularly relevant for high-stakes environments like aviation.

#### 3.2.3 Challenges

EEG provides promising opportunities and valuable metrics for assessing MF. However, despite its potential, analytical approaches including event-related potentials (ERPs), spectral analyses, and time-frequency domain analyses face significant challenges when applied in real-world settings. A key issue is the limited specificity in frequency indices associated with cognitive decline. Many markers commonly linked to MF and performance decrements also occur in other cognitive states—such as elevated mental workload, stress, or drowsiness—making it difficult to isolate neural signatures uniquely related to MF. Furthermore, the temporal instability of spectral markers and the dependence of ERP-based measures on precisely time-locked events complicate their use for real-time monitoring. As a result, these traditional methods provide only discrete snapshots of MF rather than continuous assessments, restricting their applicability in dynamic operational environments (Dehais et al., 2020; Roy et al., 2013, 2016).

Beyond interpretational issues, EEG signals are also highly susceptible to artefacts that hinder reliable fatigue assessment in real-world conditions. Physiological artefacts—including muscle activity, eye blinks, and neck muscle contractions—can distort recordings, particularly in frontal and temporal regions, and tend to increase as fatigue progresses. Motion artefacts from head and body movements further compromise signal stability, especially in dynamic environments such as flight simulators or real aircraft, where electrode displacement may lead to signal loss. External noise sources—power line interference, electromagnetic noise from avionics, and vibrations in flight—can additionally contaminate EEG signals, particularly in lower-frequency bands. To mitigate these issues, advanced signal processing techniques such as independent component analysis (ICA) and artefact subspace reconstruction (ASR) are typically employed. ASR, in particular, has been shown to efficiently remove noise while preserving neural activity, enabling reliable brain-state estimation with dry EEG in real-flight conditions (Callan et al., 2018; Dehais et al., 2019). However, implementing these methods in real-time applications remains difficult due to computational constraints and the need for robust, low-latency processing.

### 3.3 Functional near-infrared spectroscopy

**Figure 1:**
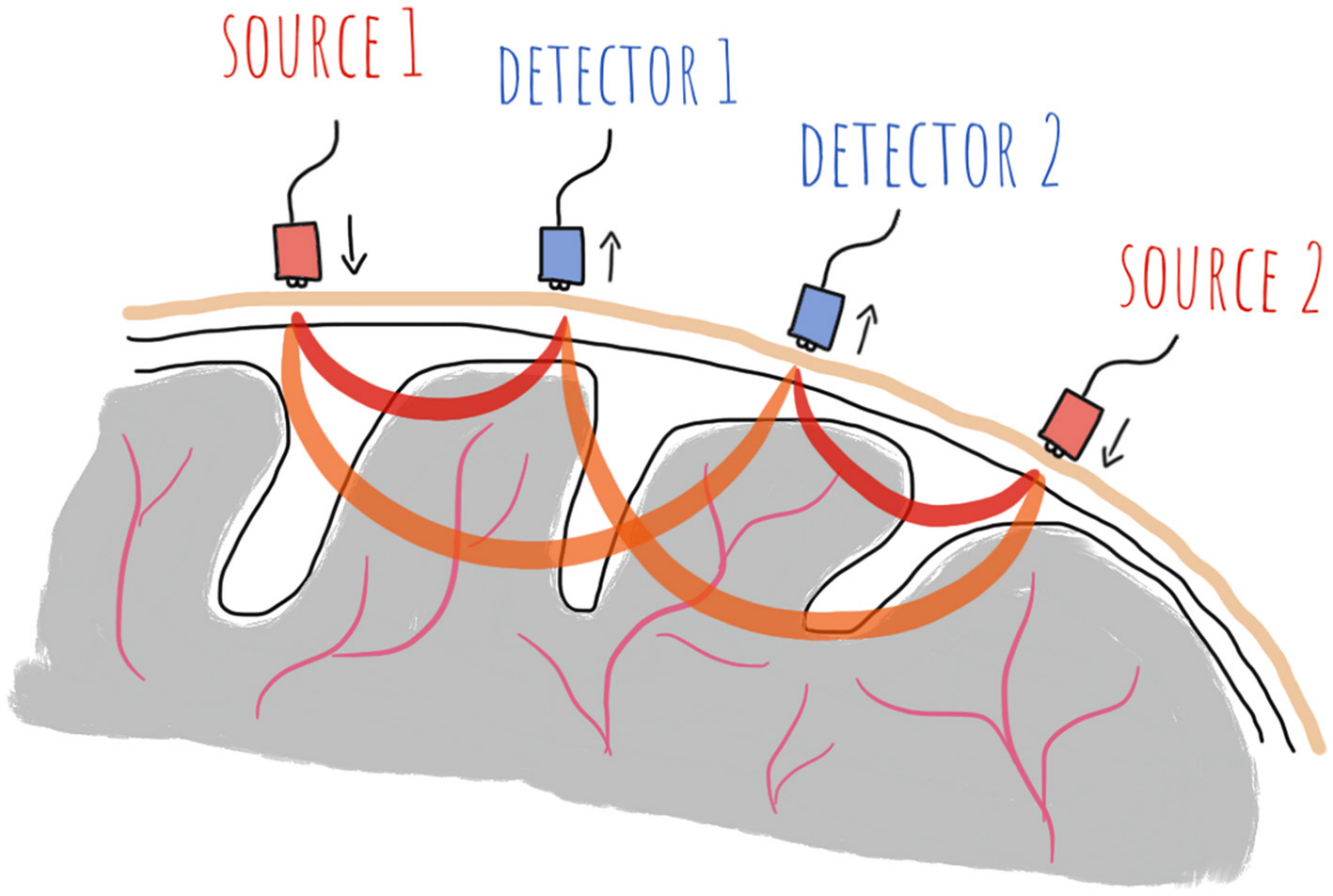
Visualisation of the banana-shaped path of the light through the brain from a light source to a detector during an fNIRS measurement (Hamann, 2023, p. 5).

Functional near-infrared spectroscopy (fNIRS) is a non-invasive method for measuring stimulus- or task-induced changes in the oxygen consumption in the cortical tissue (Huppert et al., 2009). Increasing cortical activity, for example due to higher task demands, leads to an increase in oxygen consumption in the involved areas, and the resulting higher demand for oxygen is compensated for by an increasing blood flow through the tissue. An increasing amount of oxygenated blood (HbO) is transported to the active area, while deoxygenated blood (HbR) is simultaneously “washed out” (Huppert, 2016; Huppert et al., 2009). HbO and HbR have different light-absorbing properties, which make an fNIRS measurement possible. During such a measurement, light in two different wavelengths in the near-infrared spectrum (between 650 and 900 nm) is shone into the brain by placing a light source like an LED or laser onto the scalp. The light permeates the skull and tissue until it reaches the cortex, is scattered and absorbed on its approximately banana-shaped path through the brain and reaches a detector that is placed a few centimetres apart from the light source, see Figure 1.

The difference between the light intensity upon entry into and exit from the brain is measured, and by converting into HbO and HbR concentrations using the modified Beer-Lambert law (MBLL) (Huppert et al., 2009; Jacques, 2013), changes in cortical oxygenation, and thus in brain activity, can be detected (Bae, 2015; Huppert, 2016). The light sources and detectors, both called optodes, are kept in place with a flexible cap like the ones used for EEG, or a headband for prefrontal measurements. The farther apart the source and detector, the deeper the light penetrates the brain. With an optimal distance of around 3 cm between source and detector, the light can reach approximately 1–2 cm deep into the cortex (Huppert, 2016).

fNIRS devices are wearable and can be used as a single measurement or in combination with EEG. The fNIRS signal is usually sampled at around 2–4 Hz due to the natural slow change in the haemodynamic signal underlying the method, but it can achieve a spatial precision of about 2–3 cm (Huppert, 2016). fNIRS data are rather robust against contamination with electrical noise from the recording environment or from movement artefacts (Liu et al., 2015), making it an ideal candidate measurement for applied settings like the cockpit.

#### 3.3.1 Measuring mental fatigue using fNIRS

In fNIRS, changes in cortical activation are measured by analysing changes in HbO and HbR (and sometimes by calculating the total concentration of haemoglobin, HbT). Various measures have been used for this purpose, of which this chapter will provide an overview.

##### 3.2.1.1 Moments of distribution

The most common way of analysing fNIRS data is computing and comparing moments of distribution, like the mean or peak concentrations of HbO and HbR, or the skewness, kurtosis or variance of the data in a given time interval or experimental condition. These parameters are often used in basic and applied tasks to indicate changes in cortical oxygenation. However, these metrics are rather unidimensional, require quasi-stationary signals and provide only limited information in tasks with longer durations and varying demands in which frequent fluctuations of cognitive activity are expected.

##### 3.2.1.2 Power spectra

Similar to EEG data, fNIRS data can also be transformed into the frequency domain. While this approach is less common than using moments of distribution, it has been applied to MF assessment (Chuang et al., 2018).

##### 3.2.1.3 Connectivity

Connectivity describes the co-variation of the fNIRS signal within or between certain cortical areas and is indicative of the distribution of cortical processes involved in the activity. Parameters used for this kind of analysis are for example Pearson's correlation coefficients (e.g., Badarin et al., 2024) or wavelet-based measures like the wavelet transform and wavelet-based coherence (L. Xu et al., 2017).

##### 3.2.1.4 Cortical lateralization

Lateralization describes to which extent processes are distributed across the two brain hemispheres. There is first evidence that this method can be used in the context of assessing MF using fNIRS (Zhang et al., 2017).

##### 3.2.1.5 General linear models

Another way of analysing fNIRS data is a regression-based estimation of the cortical activation. In this approach, based on the hypothesis that the fNIRS data follow a haemodynamic response function, said function is modelled into the regression. Beta coefficients of the regression are obtained for each channel (or region of interest) and can be compared between experimental conditions or over time. While successfully showing activation changes between different levels of mental workload, the application of this approach to MF paradigm has yielded mixed results (Hamann and Carstengerdes, 2023; Nogueira et al., 2022).

##### 3.2.1.6 Steady-State Visual Evoked Potentials (SSVEPs)

While SSVEPs as measures of attention and vigilance are well-researched in EEG, there are only a few studies yet in which the technique has been applied to fNIRS. Wang et al. (2020) used flickers with a frequency of 0.2 Hz and showed corresponding peaks in the frequency-domain transformed fNIRS signal at 0.2 and 0.4 Hz, thus proving the feasibility of SSVEPs in fNIRS research. Li et al. (2016) used fNIRS data to identify if participants focussed on presented stimuli in order to improve SSVEPs in EEG. Meng et al. (2023) combined fNIRS and EEG data to improve their SSVEP measurement, and could thereby reduce the pixel density of the flickers to 20%, thus increasing participants' comfort. These findings, while not directly related to MF research, show that the SSVEP paradigm can be used on, and possibly improved with, fNIRS data.

#### 3.3.2 Empirical findings

The body of empirical findings on fNIRS-based MF assessment is growing steadily, although the findings are mixed. Studies have found increasing cortical activation with increasing time on task and thus growing MF. Mean HbO increased in prolonged simulated driving tasks of 3–7 h (Li et al., 2018, 2009), and in realistic tasks in the medical field lasting up to 5 h (Nihashi et al., 2019), or at least trends of increasing HbO during simulated drives of up to 7 h (Li et al., 2024b,c). Moreover, peak oxygenation (HbO) was found to increase in a visuospatial 2-back task with time on task until around 45 min, then decline again until minute 60 (Karthikeyan et al., 2022). In a similar fashion, an increase in power spectra in HbO has been found to coincide with EEG alpha band increases after 60 min of simulated driving (Chuang et al., 2018). However, there are also contradictory findings of significantly reduced mean HbO in fatigued vs. alert states in basic PVT tasks (Nogueira et al., 2022) and simulated driving (Nguyen et al., 2017). Finally, Hamann and Carstengerdes (2023) induced MF via an adapted auditory 1-back task combined with a visual monitoring task over the course of 90 min in the context of a flight simulation, but found no consistent trend in cortical activity in HbO or HbR.

Increasing MF generally seems to decrease connectivity between brain regions and change the distribution of activity between the two hemispheres. Badarin et al. (2024, 2022) used a Sternberg task to induce MF and reported decreasing connectivity between brain regions. Similar findings were reported by Li et al. (2024a) after a 110-min version of the U-K test with and without a secondary auditory task, and by Peng et al. (2021) in a complex paradigm combining PVT with arithmetic tasks, reading in a foreign language and simulated driving (a re-analysis of their data can be found in Yan et al., 2024). Xu et al. (2017) found decreasing connectivity after 1 h of driving combined with a secondary mental arithmetic task. Zhang et al. (2017) compared lateralization of cortical activity in a 20-min CPT before and after an 80-min verbal 2-back task for MF induction and found increasing lateralization in the right cortex.

Moreover, classification algorithms trained on various parameters like mean, variance, skewness or kurtosis could be used to successfully identify MF. Varandas et al. (2022) combined a visuospatial working memory task with a concentration task that required mental arithmetic, and a digital lesson including a reading comprehension test. Pan et al. (2022) and Dehais et al. (2018) utilized realistic flight simulations with either time of day (Pan et al., 2022) or a secondary auditory oddball task (Dehais et al., 2018) as MF induction. In all three approaches, MF could be assessed successfully.

#### 3.3.3 Challenges

Because fNIRS is a comparatively new method, especially for applied research, few standardised procedures exist for data pre-processing and analysis. Like EEG, fNIRS is not free from artefacts. Bright light sources or infrared radiation should be avoided, for example by shielding detectors with an additional dark cap. Moreover, the fNIRS signal can be affected by systemic artefacts such as heart rate, respiration, and body movements that may displace sensors or interfere with cerebral blood flow (Brigadoi et al., 2014). Thus, the data must be cleaned before further processing, e.g., by means of filtering or regression-based statistical methods. Accordingly, robust, real-time pre-processing algorithms are still needed. Even with cleaned data, the optimal parameters for MF detection remain debated. The choice between analysing oxygenated (HbO), deoxygenated (HbR) or total haemoglobin (HbT) concentrations, combined with widely differing analysis strategies and measurement locations (a discussion of which is omitted in this report for the sake of brevity), makes it difficult to compare literature and identify consistent patterns of MF development in fNIRS data. Moreover, only a few studies have focused on gathering and analysing continuous data, most relying instead on pre-post comparisons. Thus, information about changes in fNIRS data over the course of experiments is still rare, limiting our ability to determine whether fNIRS can reliably assess MF continuously. In sum, the literature highlights the need for further research—particularly regarding the sensitivity, reliability and validity of different fNIRS parameters for the continuous MF assessment.

### 3.4 Eye-tracking

Eye-tracking offers a “behavioural lens” into the transition from alertness to fatigue by capturing measurable changes in eye movements and physiological signals. As MF progresses, it affects ocular behaviours in ways that are both observable and quantifiable. Eye-trackers utilize cameras or sensors to record these parameters, often in real-time, offering a non-intrusive method to assess cognitive and emotional states. Eye-trackers can be broadly categorized based on their underlying technology and application context, including video-based eye trackers, electro-oculography (EOG) systems, and wearable eye trackers. Video-oculography or video-based eye trackers use cameras and infrared light to track the position of the pupil and corneal reflection. These systems can be mounted on monitors or integrated into devices, making them suitable from stationary setups in lab experiments to virtual reality applications. Wearable eye-trackers, such as head-mounted systems or smart glasses, enable eye-tracking in dynamic, real-world environments. However, increased mobility often leads to decreased accuracy of measures (Carter and Luke, 2020; Martinez-Marquez et al., 2021). Electro-oculography (EOG) measures electrical potentials around the eyes to track movement, functioning well in conditions where direct visual line-of-sight is challenging (Tian and Cao, 2021). While less precise than video-based systems, EOG is robust to lighting changes and has been applied in fatigue studies. Currently, most eye-trackers are based on video. However, with technological advancements, eye-tracking systems have become more robust, cost-effective, accurate, and less intrusive, making them increasingly accessible for real-time fatigue state monitoring in operational setting (Martinez-Marquez et al., 2021).

#### 3.4.1 Measuring mental fatigue using eye-tracking

MF manifests in measurable ocular behaviours. Parameters such as blink rate, saccadic dynamics, fixation patterns, and pupil responses have been studied extensively as indicators of cognitive and emotional states. A number of the derived eye tracking parameters are useful MF indicators.

##### 3.4.1.1 Pupil dilation and variability

Mental fatigue is often related to a decrease in baseline pupil diameter or decreased pupil diameter variability. Moreover, lower dilation speeds are linked to a decrease in alertness (Bafna and Hansen, 2021).

##### 3.4.1.2 Blinks

Increased blink count (number of blinks in a trial), frequency (number of blinks in a trial divided by the time) and normalized blink ratio are associated with an increase in MF, boredom and reduced vigilance (Bafna and Hansen, 2021).

##### 3.4.1.3 Saccade and micro saccades

Saccades are rapid eye movements that shift the gaze between points of interest. Microsaccades are tiny, involuntary saccades that occur during fixation to prevent sensory fading and enhance visual acuity. Mental fatigue slows down saccade velocity, reduces saccade amplitude, and increases saccade duration. Microsaccades may become less frequent or erratic during prolonged mental exertion (Bafna and Hansen, 2021).

##### 3.4.1.4 Ocular drift

Ocular drift refers to the small, slow, involuntary eye movements that occur during fixation, often not perceivable to the observer. Mental fatigue can increase the amplitude and irregularity of ocular drift, reflecting diminished oculomotor control and reduced focus stability (Di Stasi et al., 2013).

##### 3.4.1.5 Fixation patterns

Gaze fixation refers to the period during which the gaze remains stable on a specific point of interest. Fixation patterns are the spatial and temporal characteristics of these pauses. Mental fatigue leads to longer fixation durations and fewer fixations overall (Xu et al., 2018).

##### 3.4.1.6 Percentage of eye closure (PERCLOS)

PERCLOS measures the percentage of time the eyes are at least 80% closed over a given period. Increased PERCLOS is a strong indicator of MF, particularly in tasks requiring sustained attention (Bafna and Hansen, 2021).

#### 3.4.2 Empirical findings

In eye-tracking experiments on MF, participants are often required to perform vigilance tasks, such as monitoring a static screen for subtle changes or responding to infrequent stimuli. This is not just true for basic experimental tasks but also for realistic simulations which often require sustained attention, such as prolonged driving or flights (Ma et al., 2018; Qin et al., 2021; Rosa et al., 2022). Prolonged engagement generally leads to decreased gaze fixation times, reduced pupil size and pupil size variability, and increased blink frequency (Hopstaken et al., 2015; Martin et al., 2022; Naeeri et al., 2021; Unsworth et al., 2024; Zhao et al., 2025), even though there is also evidence of effects in the opposite direction (Hu and Lodewijks, 2021; Lampe and Deml, 2022). These eye behaviour patterns align with declining cognitive resources and attentional control. Machine learning algorithms have proven useful for MF assessment and even show potential for real-time monitoring: the combination of eye-tracking metrics with other physiological data (heart rate variability) have proven effective in assessing MF (Qin et al., 2021). Savas and Becerikli (2018) proposed a real-time driver fatigue detection system based on the Support Vector Machine (SVM) algorithm and measures of PERCLOS and blinks, combined with facial expressions, to detect signs of MF. Makhmudov et al. (2024) developed a system that uses convolutional neural networks (CNNs) to analyse gaze and yawning behaviour, achieving over 96% accuracy in detecting MF indicators from a web camera. Xu et al. (2018) showed the potential of using fixation time and pupil area metrics and the “fuzzy K-nearest neighbour” algorithm to assess MF during monotonous driving simulations in real time.

#### 3.4.3 Challenges

Despite these advancements, several challenges remain in leveraging eye-tracking for MF monitoring. One of the main challenges is variability in individual gazing behaviour, which can lead to inconsistencies in MF assessment models (Zargari Marandi et al., 2018). Differences in baseline pupil size, blink rates, and gaze behaviour necessitate personalized calibration for optimal accuracy. Furthermore, environmental factors such as lighting conditions, screen glare, and occlusions from eyeglasses or head movements can impact data reliability (Adão Martins et al., 2021; Vrzakova and Bednarik, 2012). Addressing these challenges will require the development of adaptive algorithms that can account for individual differences and external influences in real-time applications (Li and He, 2024), and/or the need to train the systems for each pilot individually.

## 4 Conclusion: bridging the gap between laboratory and cockpit

The aim of this work was to explore and compare multiple head-worn sensing methods for monitoring mental fatigue (MF), and to discuss the methodological and practical challenges that must be addressed to enable pilot-state monitoring in aviation. The review began by summarising key research paradigms and highlighting the strengths and limitations for each approach. But how large is the gap between the current state of research and an application in the cockpit?

Subjective measures are easy, quick, and cost-efficient and have therefore been widely used to assess MF. Yet, they suffer from well-known limitations, including intrusiveness, low temporal resolution and dependence on the pilot's self-assessment and honesty. Thus, these methods do not qualify for continuous MF monitoring in the cockpit. Consequently, research has turned to behavioural and physiological measures such as EEG, fNIRS and eye-tracking to capture the pilot's state. Yet, to date there is still no viable, operational pilot monitoring system in place. Why is that and is it justified to continue this line of research?

EEG has been used for decades to assess cognitive performance in both controlled laboratory settings and real-world environments, including aircraft. EEG provides high temporal resolution and direct neural markers of cognitive states. Various EEG metrics—such as time-domain analysis, spectral analysis, time-frequency decomposition, and connectivity measures—have been extensively validated and proven effective in tracking mental states such as MF. Recent research leveraging “invisible flickers” for SSVEPs highlights their potential for assessing attentional engagement in pilots and evaluating the cognitive processing required to maintain situation awareness. By flashing different cockpit elements at distinct frequencies (e.g., the speed indicator, altimeter, or Flight Mode Annunciator panel), it may be possible to determine how effectively MF affects pilot performance (Dehais et al., 2019). This approach holds great promise for studying the depth of information processing in the cockpit and could serve as a valuable complement to eye-tracking systems. While eye trackers primarily measure visual fixation and gaze direction, SSVEPs can capture peripheral visual attention, also known as “covert” attention—a crucial skill for pilots when supervising complex flight decks. This capability makes SSVEPs particularly useful for assessing attentional shifts and the processing of visual information beyond direct gaze, offering deeper insights into pilot's cognition that eye trackers alone cannot provide.

fNIRS measurements are less prone to noise and artefacts commonly found in a cockpit, such as movement and electrical noise, than EEG data, and the higher spatial resolution could make miniaturization with only a few channels possible. However, the method remains highly sensitive to infrared light exposure from the sun and G-forces, particularly in real-flight conditions, and only few studies have focused on continuous fNIRS measurements for gradual MF increases. No “gold standard” parameter has yet emerged, but fNIRS's ability to detect changes in cortical activity makes it a promising candidate for tailored assistance, particularly for detecting dwindling attention or increasing MF. The first studies have shown its applicability both in flight simulations and real flights. Advancements in eye-tracking technology for MF detection have shown significant potential for real-time, non-intrusive monitoring. Eye-tracking provides valuable data on gaze behaviour and attentional shifts but is limited in its ability to infer deeper cognitive processes beyond visual fixation. It provides a range of metrics for detecting MF, including blink rate, pupil dilation, and PERCLOS. One of its key strengths is the use of remote, video-based eye-tracking systems, which offer a non-intrusive solution that does not interfere with the pilot's tasks or field of vision. Advances in machine-learning-based fatigue detection have shown promise in controlled real-time simulation environments.

The individual measurements offer unique advantages but also inherent limitations that affect cockpit applicability. A multimodal approach—integrating EEG, fNIRS, eye-tracking, and situational or aircraft data—could present a promising solution for improving the robustness and redundancy in MF assessment in aviation (Boumann et al., 2023; Hamann and Carstengerdes, 2024; Hinss et al., 2022). To move beyond single-sensor limits, event-locked multimodal fusion ties ocular events to brain responses so we infer not just *where* the pilot looks but *how deeply* information is processed. For EEG and eye-tracking, time-lock EEG to fixation onsets on safety-relevant AOIs to compute fixation-related potentials (EFRPs) and brief spectral changes—capturing processing depth and time-on-task drift (Degno and Liversedge, 2020; Takeda et al., 2001). Also time-lock to blinks to extract blink-related oscillations (BROs), which are sensitive to task/sensory context (Liu et al., 2019) and have been shown to vary with cognitive load in a flight-like multitasking environment (MATB-II; Page et al., 2024). For fNIRS + eye-tracking, treat fixations as GLM events to estimate fixation-triggered ΔHbO/ΔHbR—as demonstrated in fixation-related fNIRS during natural reading (Roelke et al., 2020)—a procedure that can be ported to cockpit AOIs to test whether safety-relevant glances reflect deeper cortical processing. Finally, EEG-fNIRS hybrids already demonstrate complementary electro-/hemodynamic MF markers in realistic driving and flight contexts—an architecture that extends naturally with gaze events for cockpit-grade state estimation (Chuang et al., 2018; Dehais et al., 2018). Yet, synchronizing data from different modalities is complex and requires advanced algorithms capable of processing large volumes of data in real-time without compromising performance or accuracy (Wang, 2024).

Bridging the gap from laboratory studies to operational flight also involves substantial technological and practical challenges. Most research to date has focused on group-level analysis and provides results for the average individual. Whereas, tailored assistance based on a pilot's current cognitive state requires accounting for inter- and intraindividual differences. Personal calibrations—and potentially frequent recalibrations—will likely be necessary to accommodate fluctuations in brain activity over days and weeks.

In most studies discussed in this paper, data are processed *post-hoc* and real-time implementation in operational flight conditions remains especially difficult for EEG, which is highly sensitive to electronic and electromagnetic noise, speech and motion artefacts. For cockpit applications, raw physiological data must be processed in real-time, thus requiring even more hardware and software to be integrated into the cockpit. Despite recent advancements in signal processing, the authors are not aware of any hardware or software that fits these requirements fully which could be integrated into a cockpit.

From a practical standpoint, neither EEG nor fNIRS is contactless and both require the pilot to wear a tight cap or headband throughout the flight. In commercial aviation where pilots do not to wear a helmet, the integration of EEG or fNIRS sensors poses a challenge in terms of ease of use, acceptance and comfort. The devices need to be unobtrusive, wearable, comfortable and easy to apply and remove by pilots (Kneffel et al., 2025). In addition, the hardware must not limit the pilots' field of view or movement. The device must be fast and easy to remove in case of danger or evacuation, and must not pose a safety hazard due to inflammable batteries. Most of the measurements are undertaken in laboratory settings with cumbersome devices that tend to become uncomfortable when worn for a prolonged period of time (see e.g., Radüntz and Meffert, 2019 for an analysis of EEG device wearing comfort). However, miniaturisation is advancing rapidly. Some recent devices integrate EEG sensors into standard audio headsets or mount eye-tracking cameras directly in the cockpit, suggesting that intrusiveness will continue to decrease. Finally, integrating physiological monitoring into the cockpit—a pilot's workplace—raises important questions of data protection, ethics, cyber-security, and certification for use in aircraft. These regulatory and ethical frameworks must evolve in parallel with technological developments and be tailored to specific operational contexts. In sum, there is a need for miniaturized portable, comfortable, safe, secure and trustworthy systems that still offer excellent signal-to-noise ratios and high data quality across diverse conditions.

Assessing MF in operational aviation remains a critical challenge due to the complex and dynamic nature of real-world flight conditions. Yet, only with reliable and valid measurement methods will an operator state assessment provide a benefit to the pilot. Such an assessment will need to be very precise and leave no room for false detections and resulting unsuitable adaptations. When deciding for (single or combined) sensors, care should be taken that are and remove by pilots (Kneffel et al., 2025).

Overall, there is a clear need for miniaturized, comfortable, safe, and trustworthy systems that can deliver high-quality data across a wide range of flight conditions. Assessing MF in operational aviation remains challenging due to the complexity of real-world conditions, yet reliable and valid measurement methods are essential for operator-state assessment to provide real benefit to pilots.

Progress will not occur through a single leap from laboratory to cockpit. Instead, following Peysakhovich et al. (2018), we recommend a staged approach, which outlines four key phases for the integration of neurophysiological monitoring in aviation:

- Stage I: Validate metrics in controlled settings: Pilot Training and Flight Performance Analysis on Ground—Initial validation of multimodal neurophysiological metrics in controlled environments, ensuring reliable MF detection and assessing their impact on pilot training and cognitive performance.
- Stage II: Integrate recordings for flight data augmentation: On-Board Recordings for Flight Data Augmentation—Implementation of neurophysiological recordings as supplementary data sources for the aircraft's “black box”, providing deeper insights into pilot cognitive states during flight.
- Stage III: Enable adaptive alerting systems: Flight Deck Adaptation with Warning and Alerting Systems—Real-time monitoring of pilot cognitive states to enable adaptive warning systems that, for example, support situation awareness and prevent performance degradation due to MF.
- Stage IV: Advance AI-driven automation: Multimodal-Based Aircraft Adaptation and Automation—Advanced AI-driven systems utilizing multimodal neurophysiological data to enhance pilot-aircraft interaction, with the long-term goal of enabling adaptive automation, where the aircraft could temporarily take over control in cases of pilot incapacitation.

The gap between laboratory-based pilot-state monitoring and cockpit implementation remains substantial—but so too does our expanding understanding of human cognition and neurophysiology.It is essential to be aware of the current capabilities and limitations of methods, the challenges the real world poses in comparison to a controlled laboratory environment, and to derive the focus of research activities from this knowledge. Pilot-state monitoring is far from a solved problem, yet it is a solvable one—provided that the field embraces incremental, evidence-based progress toward safe, practical, and accepted integration in aviation.
